# Clinicopathological correlation of psychosis and brain vascular changes in Alzheimer’s disease

**DOI:** 10.1038/srep20858

**Published:** 2016-02-12

**Authors:** Simon Kang Seng Ting, Ying Hao, Pei Shi Chia, Eng-King Tan, Shahul Hameed

**Affiliations:** 1 Department of Neurology, Singapore General Hospital, Singapore, Singapore; 2 National Neuroscience Institute, Singapore, Singapore; 3 Health Services Research and Biostatistics Unit, Division of Research, Singapore General Hospital, Singapore, Singapore

## Abstract

Psychosis is common in Alzheimer’s disease (AD). However, studies on neuropathology in vascular etiology contributing to psychosis in AD is lacking to date. The aim of this study was to investigate neuropathological vascular related changes in Alzheimer’s disease with psychosis. Data of patients with AD from the National Alzheimer’s Coordinating Center between 2005 to September 2013 was accessed and reviewed. Presence of psychosis was determined based on Neuropsychiatric Inventory Questionnaire taken from the last visit within one year prior to death, and patients were divided into psychosis positive and negative group. Comparison of clinical details and neuropathological vascular changes between the groups was performed using Wilcoxon rank sum test and Chi-square/ Fisher’s exact test. Significant variables were further included in a multivariate logistic model. Overall, 145 patients was included. Of these, 50 patients were psychosis positive. Presence of one or more cortical microinfarcts and moderate to severe arteriosclerosis was found to be positively associated with psychosis. Our results suggest vascular changes correlate with psychosis in Alzheimer’s disease.

Psychosis is encountered in Alzheimer’s disease (AD), and common psychotic symptoms observed are delusions and hallucinations. Wragg and colleagues^1^ reviewed 30 studies and found that approximately 30–40% of Alzheimer’s disease patients had delusions at some point during their illness, 28% had hallucinations, and nearly 35% had other psychotic symptoms that were difficult to categorize. In another comprehensive review of clinical studies involving 9,749 subjects and 55 studies, the median prevalence of AD with psychosis was 41.1% (range = 12.2–74.1%), with a 3-year cumulative incidence approximating 50%^2^,^3^. Epidemiologic studies have found a lower point prevalence of AD with psychosis, closer to 25%^4^. These differences may reflect that the rate of AD with psychosis is dependent on AD stage, with low rates of psychosis in prodromal and early AD and higher rates in middle and later stages^5^,^6^,^7^. Meanwhile, it also has been shown that accelerated accumulation of hyperphosphorylated microtubule-associated tau protein is associated with clinical psychosis in AD^7^.

Vascular etiology contributing to clinical psychosis has been described in literature; for instance, cortical or hemispheric infarcts are reported to be associated with psychosis^8^. Radiological evidence of subcortical ischemic lesions also has been implicated with late-onset psychosis patients^9^,^10^. However, in AD patients with psychosis, MRI studies on white matter hyperintensities have only shown inconsistent results. Lee and colleagues noted white matter changes were not associated with paranoid delusion and hallucination, but only with delusional misidentification^11^. Meanwhile, Barber and colleagues noted absence of occipital WMH was associated with delusions and visual hallucinations^12^. Furthermore, Howanitz and colleagues noted no significant difference of white matter intensity between delusional and non-delusional groups of AD^13^. At the same time, detailed neuropathological study in vascular etiology contributing to psychosis in AD also largely remains lacking to date.

This study aimed to investigate neuropathological findings, especially vascular-related changes, in Alzheimer’s disease with psychosis. We analyzed participants included in the National Alzheimer’s Coordinating Center (NACC) database who fulfilled a diagnosis of definite AD for high likelihood of dementia due to AD. We postulated that vascular brain changes and burden are likely to be positively correlated to psychosis in AD.

## Methods

### Study design and participants

This study accessed NACC neuropathology data from all patients included in the NACC Neuropathology Data Set. The NACC collects data from National Institute on Aging (NIA) –funded Alzheimer Disease Centers in the United States and the data collection process is in accordance to NIA policies. Clinical information was crossed-referenced and abstracted from the NACC Uniform Data Set (UDS)^14^. The NACC study population is clinic-based, and UDS includes subjects with a range of cognitive status—normal cognition, MCI, and demented. Each center enrolls its subjects according to its own protocol. Subjects may come from clinician referral, self-referral by patients or family members, active recruitment through community organizations, and volunteers who wish to contribute to research on various types of dementia^15^.

Patients with definite AD (based on NIA/Reagan Institute neuropathological criteria) or high likelihood of dementia due to AD^16^ were included in this study. Information on baseline visits and follow-up visits prior to death were reviewed. Only patients with no Lewy Body pathology identified, based on criteria of Consortium of Dementia with Lewy Bodies^17^ (reflected on neuropathology dataset item 13A), were included into the study. Patients without follow-up data within one year of death were excluded from the study. Research using the NACC database is approved by University of Washington Institutional Review Board, and authors who access the data are required to sign and comply with the data use agreement. Written informed consent was obtained from all participants and informants with their anonymity preserved. The study and use of the data was also approved by authors’ home institutional review board Singhealth.

### Neuropsychiatric Inventory Questionnaire (NPI-Q)

The presence of psychosis was based on available information reflected in the Neuropsychiatric Inventory Questionnaire (NPI-Q)^18^, specifically item 5 of the NACC Uniform Data Set. The NPI-Q information utilized for analysis was based on assessment during the most recent visit, performed within one year prior to patient mortality. Psychosis was considered positive if patient exhibited delusions and/or hallucinations (form 5B, questions 2a, 3a) in a one-month time frame prior to assessment.

Variables from the neuropathological data in the dataset that analyzed and compared AD patients with and without psychosis were incorporated and summarized in Table 1.

**Figure 1:**
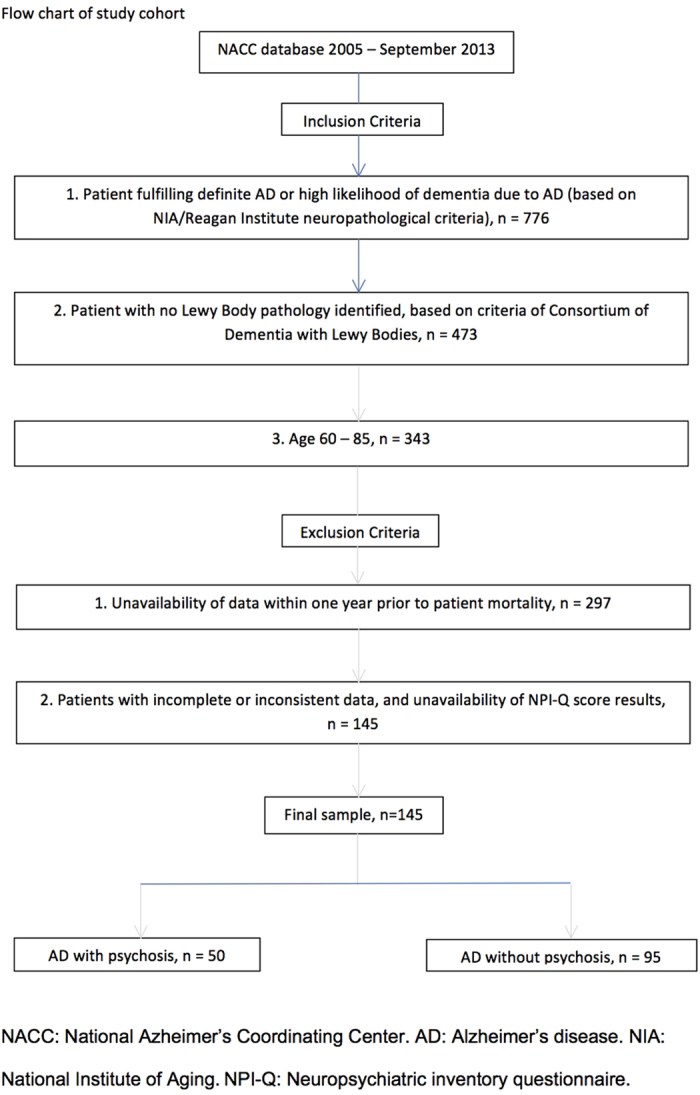
Flow chart of study cohort.

Another inclusion criterion was the age range of 60 to 85, and exclusion criteria also included incomplete or inconsistent data of demographic details and lacking of Neuropsychiatric Inventory Questionnaire (NPI-Q) score results. For instance, patients with MMSE recorded as ‘96’ were excluded from the cohort. The summary details of the inclusion and exclusion criteria for the study cohort have been included in Fig. 1.

### Statistical methods

Psychosis and non-psychosis groups were compared using Wilcoxon rank sum test with continuity correction for continuous variables and Chi-square /Fisher’s exact tests for categorical variables. P-values of less than 0.05 were considered statistically significant. Variables with p-value of less than 0.2 in the univariate analysis were further included in a multivariate logistic model. While by applying convention p-value of 0.05 in selecting variables from univariate analysis may fail to identify important variables in multiple variables analysis, the decision of cut-off p-value is usually determined by the statisticians. For instance, Budtz-Jørgensen and colleagues as well as Zoran Bursac and colleagues recommended p value of 0.2 and 0.25 respectively^19^,^20^. The sample size also affects the decision of the number of variables included in the multiple logistic models. Our current study decision to employ cut-off value of 0.2 is based on general concept that with sample size of 50 psychosis patients, including 4–5 variables in the model will be appropriate. All statistical analysis was performed in R software (Version 3.0.2).

## Results

The final dataset analyzed for the current study included 145 patients evaluated at National Institute on Aging–funded Alzheimer Disease Centers (ADCs) from 2005 to September 2013. The NACC database includes 34 past and present ADCs.

Both groups of patients were comparable for baseline demographics. Figure 1 summarizes the algorithm of how current cohort was arrived.

Presence of one or more cortical microinfarcts (p = 0.041) and moderate to severe arteriosclerosis (p = 0.002) were positively associated with psychosis. Table 1 summarizes the demographical characteristics and neuropathological variables results of the patients. In the multivariate logistic regression analysis, using psychosis status as dependent variable and adjusted by education years and presence of amyloid angiopathy, the p-value of the independent variables of both ‘presence of one or more cortical microinfarcts’ (adjusted odds ratio: 3.85, 95% confidence interval: 1.29–12.24, p = 0.018) and ‘moderate to severe arteriosclerosis’ (adjusted odds ratio: 3.46, 95% confidence interval: 1.63–7.55, p = 0.001) remained significant, suggesting a positive correlation between the two.

Table 2 summarizes the NPI-Q results of Alzheimer’s disease patients with and without psychosis.

## Discussion

Previous studies on psychosis in AD, particularly before 1990s, have been limited by few important factors. These included inconsistent criteria for AD, uneven sample size, and the lack of operational definitions for psychosis^21^. Furthermore, the prevalence of psychosis usually varies in the course of AD, and some studies noted that the severity of cognitive impairment was positively associated with the presence of psychosis. The results suggested that in general, the prevalence of psychosis increased as the level of cognitive impairment advanced^21^. Hence, timeline selection of psychosis analysis during the course of AD poses further challenge for studies in this topic.

Another important factor to consider for study involving psychosis is the concurrent presence of Lewy body pathology in the patients. Lewy body pathology likely contributes to psychosis in AD^22^,^23^. In clinical practice, confirmatory judgment of its concurrent presence would not be possible purely based on clinical approach without assistance of biomarkers, which is usually only performed under research protocol. Generally, its presence is only made known during post mortem study. Due to these reasons, performing a well-controlled clinical study on psychosis in AD would not be an easy task. This probably explains why radiology studies to date have never been able to yield a consistent result^11^,^12^,^13^. In order to minimize these confounding factors, we only included patients without Lewy body pathology, and matched best for demographics, cognitive status and visit timing, which was within one year period before patient mortality.

A previous study that derived from NACC data and analyzed the relationship of clinical factors and apolipoprotein E status with psychosis in AD failed to show a significant relationship between diabetes and hypertension with psychosis in multivariate analysis. The authors interpreted conservatively that an increased vascular burden was likely not associated with psychosis in AD. However, the interpretation was derived from indirect findings and without any neuropathological data^24^.

This study found that the presence of one or more cortical microinfarcts being positive correlated with psychosis. This is in line with previous studies that suggest cortical lesions contribute to psychosis^8^,^25^. Furthermore, some radiological studies indicate that grey matter thickness or volume is positively correlated with psychosis in AD^26^,^27^,^28^. However, the association of cortical microinfarcts with psychosis still needs to be interpreted with caution. Given that our database lacks the exact topographical distribution of the microinfarcts itself, they may have a laterality effect as suggested by previous study^8^. There are also premorbid imaging details of cortical thickness in different regions of the brain that may need to be adjusted for.

Meanwhile, we also found that the presence of moderate and severe small parenchymal arteriolar disease was positively correlated to psychosis in AD. Again, this is consistent with some radiological studies in AD that correlated white matter hyperintensities with psychosis^11^,^12^. In some non-AD studies, white matter hyperintensities were also noted to be a contributing factor to psychosis, such as late adult onset paranoid psychosis^9^,^10^.

It should be noted that the MMSE mean score was rather high for the group for mortality within one year of their assessment, as there were a significant number of patients lacking psychometric assessment scoring. We can only presume the most likely cause of missing data was due to relative advanced stage of AD and inability to complete the MMSE, as it requires a patient to cooperate with the assessment. However, the database has full details on their Clinical Dementia Rating Scale (CDR) sum score, as its administration is independent on patients’ cognitive status. We have included it in Table 1 which shows an average mean score of 12.5, corresponding to a CDR global score of 2–3^29^. This is indicative of a moderate to severe stage of disease.

Based on our findings, it is suggested that the integrity of small vessels structure at both cortical and subcortical level is likely important and correlated with psychosis in AD. The strength of current study lies in the provision of pathological evidence, which was lacking in previous studies, and fills the current knowledge gap between clinical and radiological evidence.

However, the study is limited by the retrospective nature of data collection in terms of psychotic symptoms. It was collected from the informants mainly at the time point of last contact with the physician, which could affect the reliability of data, particularly time point sampling error. Nevertheless, as psychosis is usually a recurrent symptom and tends to be more profound at the later stage of disease^18^, we believe last contact with the patients within a one year period is adequate in identifying clinical psychosis. Furthermore, since the database is mainly clinic-based, we believe proportion of patients with delirium, a significant confounding factor, should be minimal. Hence, future study should consider a more standardized study such as prospective study for better definition or identification of psychosis throughout the course of AD.

Additionally, further details such as the topographical distribution of lesions of vascular changes were unavailable for further analysis, due to the semi-quantitative nature of the data. Nevertheless, our main aim in this study was to determine the potential types of vascular changes that could correlate to AD and psychosis, in order to provide a guide for future quantitative studies.

Neuroimaging details were also not examined in this study as the focus was on the correlation of clinical psychotic symptoms and pathological changes. Future studies should look into data from neuroimaging and further correlate these with any pathological findings. For instance, cortical thickness data should be examined and adjusted before concluding cortical microinfarcts as contributing factor to psychosis.

In conclusion, our study demonstrated ischemic small vessels disease is likely associated with psychosis in AD. While cortical microinfarcts also potentially play a role in pathogenesis of psychosis, further verification study is needed.

## Additional Information

**How to cite this article**: Ting, S. K. S. *et al.* Clinicopathological correlation of psychosis and brain vascular changes in Alzheimer's disease. *Sci. Rep.* **6**, 20858; doi: 10.1038/srep20858 (2016).

## Figures and Tables

**Table 1:** Demographical Characteristics of Alzheimer’s Disease Patients and Vascular-related Variables in Patients With and Without Psychosis.

Age, mean (S.D.)	Total (n = 145)	AD with Psychosis (n = 50)	AD without Psychosis (n = 95)	p-value
	76.2 (7.1)	75.2 (7.7)	76.7 (6.8)	0.335*
Male gender, n (%)	94 (64.8)	32 (64.0)	62 (65.3)	1.000**
Caucasian race, n (%)	137 (94.5)	46 (92.0)	91 (95.8)	0.447**
MMSE, mean (S.D.)	17.4 (5.2)	16.8 (5.3)	17.7 (5.2)	0.669*
Education years, mean (S.D.)	15.6 (3.2)	16.1 (3.1)	15.3 (3.3)	0.131*
AD onset years, mean (S.D.)	8.67 (3.7)	8.78 (3.7)	8.62 (3.8)	0.881*
Psychiatric illness history, (Yes), n (%)	9 (6.2)	4 (8.0)	5 (5.3)	0.516**
On active anti-psychotic drugs, (Yes), n (%)	16 (11.0)	4 (8.0)	12 (12.6)	0.398**
CDR sum, mean (S.D.)	12.5 (4.9)	13 (4.6)	12.2 (5.0)	0.560*
VASC, (Yes), n (%)	121 (83.4)	44 (88.0)	77 (81.1)	0.404**
LINF, (Yes), n (%)	9 (6.2)	4 (8.0)	5 (5.3)	0.496**
MICRO, (Yes), n (%)	19 (13.1)	11 (22.0)	8 (8.4)	0.041**
LAC, (Yes), n (%)	21 (14.5)	7 (14.0)	14 (14.7)	1.000**
HEM (Yes), n (%)	13 (9)	5 (10.0)	8 (8.4)	0.763**
ART (Yes), n (%)	32 (22.1)	12 (24.0)	20 (21.1)	0.845**
NEC (Yes), n (%)	1 (0.7)	0 (0.0)	1 (1.0)	1.000**
SCL (Yes), n (%)	15 (10.3)	4 (8.0)	11 (11.6)	0.727**
AVAS (Moderate and Severe), n (%)	57 (39.3)	21 (42.0)	36 (37.9)	0.762**
ARTER (Moderate and Severe), n (%)	55 (37.9)	28 (56.0)	27 (28.4)	0.002**
AMY (Moderate and Severe), n (%)	66 (45.5)	18 (36.0)	48 (50.5)	0.135**

AD: Alzheimer’s disease. MMSE: Mini-mental state examination. S.D.: standard deviation. *Wilcoxon rank sum test. **Chi square/Fisher’s exact test. VASC: Is ischemic, hemorrhagic or vascular pathology present? LINF: Are one or more large artery cerebral infarcts present? MICRO: Are one or more cortical microinfarcts (including “granular atrophy”) present? LAC: Are one or more lacunes (small artery infarcts and/or hemorrhages) present? HEM: Are single or multiple hemorrhages present? ART: Is subcortical arteriosclerotic leukoencephalopathy present? NEC: Is cortical laminar necrosis present? SCL: Is medial temporal lobe sclerosis (including hippocampal sclerosis) present? AVAS: Is atherosclerotic vascular pathology (of the circle of Willis) present? ARTER: Is arteriosclerosis (small parenchymal arteriolar disease) present? AMY: Is amyloid angiopathy present?

**Table 2:** Neuropsychiatric inventory questionnaire results of Alzheimer’s disease patients with and without psychosis.

NPI-Q	Total (N = 145)	AD with Psychosis (N = 50)	AD without Psychosis (N = 95)	p-value*
Delusions, n (%)	38 (26.2)	38 (76.0)	0 (0)	<0.001
Mild, n (%)	18 (12.4)	18 (36.0)	0 (0)	1
Moderate, n (%)	13 (9)	13 (26.0)	0 (0)	
Severe, n (%)	7 (4.8)	7 (14.0)	0 (0)	
Hallucinations, n (%)	21 (14.5)	21 (42.0)	0 (0)	<0.001
Mild, n (%)	12 (8.3)	12 (24.0)	0 (0)	1
Moderate, n (%)	7 (4.8)	7 (14.0)	0 (0)	
Severe, n (%)	2 (1.4)	2 (4.0)	0 (0)	
Agitation or aggression, n (%)	71 (49)	33 (66.0)	38 (40.0)	0.005
Mild, n (%)	28 (19.3)	10 (20.0)	18 (18.9)	0.32
Moderate, n (%)	27 (18.6)	15 (30.0)	12 (12.6)	
Severe, n (%)	16 (11)	8 (16.0)	8 (8.4)	
Depression or dysphoria, n(%)	61 (42.1)	34 (68.0)	27 (28.4)	<0.001
Mild, n (%)	33 (22.8)	15 (30.0)	18 (18.9)	0.25
Moderate, n (%)	24 (16.6)	16 (32.0)	8 (8.4)	
Severe, n (%)	4 (2.8)	3 (6.0)	1 (1.1)	
Anxiety, n (%)	60 (41.4)	27 (54.0)	33 (34.7)	0.039
Mild, n (%)	25 (17.2)	11 (22.0)	14 (14.7)	0.926
Moderate, n (%)	23 (15.9)	10 (20.0)	13 (13.7)	
Severe, n (%)	12 (8.3)	6 (12.0)	6 (6.3)	
Elation or euphoria, n (%)	8 (5.5)	5 (10.0)	3 (3.2)	0.125
Mild, n (%)	6 (4.1)	3 (6.0)	3 (3.2)	0.464
Moderate, n (%)	2 (1.4)	2 (4.0)	0 (0)	
Severe, n (%)	0 (0)	0 (0)	0 (0)	
Apathy or indifference, n (%)	79 (54.5)	31 (62.0)	48 (50.5)	0.253
Mild, n (%)	28 (19.3)	8 (16.0)	20 (21.1)	0.049
Moderate, n (%)	35 (24.1)	19 (38.0)	16 (16.8)	
Severe, n (%)	16 (11)	4 (8.0)	12 (12.6)	
Disinhibition, n (%)	37 (25.5)	22 (44.0)	15 (15.8)	<0.001
Mild, n (%)	20 (13.8)	12 (24.0)	8 (8.4)	0.674
Moderate, n (%)	10 (6.9)	5 (10.0)	5 (5.3)	
Severe, n (%)	7 (4.8)	5 (10.0)	2 (2.1)	
Irritability or lability, n (%)	60 (41.4)	28 (56.0)	32 (33.7)	0.016
Mild, n (%)	27 (18.6)	8 (16.0)	19 (20.0)	0.056
Moderate, n (%)	21 (14.5)	13 (26.0)	8 (8.4)	
Severe, n (%)	12 (8.3)	7 (14.0)	5 (5.3)	
Motor disturbance, n (%)	47 (32.4)	18 (36.0)	29 (30.5)	0.629
Mild, n (%)	16 (11)	5 (10.0)	11 (11.6)	0.745
Moderate, n (%)	20 (13.8)	9 (18.0)	11 (11.6)	
Severe, n (%)	11 (7.6)	4 (8.0)	7 (7.4)	
Nighttime behaviors, n (%)	55 (37.9)	26 (52.0)	29 (30.5)	0.019
Mild, n (%)	22 (15.2)	10 (20.0)	12 (12.6)	0.938
Moderate, n (%)	24 (16.6)	12 (24.0)	12 (12.6)	
Severe, n (%)	9 (6.2)	4 (8.0)	5 (5.3)	
Appetite and eating, n (%)	49 (33.8)	23 (46.0)	26 (27.4)	0.038
Mild, n (%)	29 (20)	15 (30.0)	14 (14.7)	0.471
Moderate, n (%)	13 (9)	4 (8.0)	9 (9.5)	
Severe, n (%)	7 (4.8)	4 (8.0)	3 (3.2)	

AD: Alzheimer’s disease. NPI-Q: Neuropsychiatric inventory questionnaire.*Chi square/Fisher’s exact test.
